# Unique AGG Interruption in the CGG Repeats of the* FMR1* Gene Exclusively Found in Asians Linked to a Specific SNP Haplotype

**DOI:** 10.1155/2016/8319287

**Published:** 2016-03-02

**Authors:** Pornprot Limprasert, Janpen Thanakitgosate, Kanoot Jaruthamsophon, Thanya Sripo

**Affiliations:** ^1^Department of Pathology, Faculty of Medicine, Prince of Songkla University, Songkhla 90110, Thailand; ^2^Department of Pathology, Faculty of Medicine, Ramathibodi Hospital, Mahidol University, Bangkok 10400, Thailand

## Abstract

Fragile X syndrome (FXS) is the most common inherited intellectual disability. It is caused by the occurrence of more than 200 pure CGG repeats in the* FMR1* gene. Normal individuals have 6–54 CGG repeats with two or more stabilizing AGG interruptions occurring once every 9- or 10-CGG-repeat blocks in various populations. However, the unique (CGG)6AGG pattern, designated as 6A, has been exclusively reported in Asians. To examine the genetic background of AGG interruptions in the CGG repeats of the* FMR1* gene, we studied 8 SNPs near the CGG repeats in 176 unrelated Thai males with 19–56 CGG repeats. Of these 176 samples, we identified AGG interruption patterns from 95 samples using direct DNA sequencing. We found that the common CGG repeat groups (29, 30, and 36) were associated with 3 common haplotypes, GCGGATAA (Hap A), TTCATCGC (Hap C), and GCCGTTAA (Hap B), respectively. The configurations of 9A9A9, 10A9A9, and 9A9A6A9 were commonly found in chromosomes with 29, 30, and 36 CGG repeats, respectively. Almost all chromosomes with Hap B (22/23) carried at least one 6A pattern, suggesting that the 6A pattern is linked to Hap B and may have originally occurred in the ancestors of Asian populations.

## 1. Introduction

The cause of fragile X syndrome (FXS) is the expansion of CGG repeats in the 5′UTR of the* FMR1* gene and subsequent hypermethylation at the CpG island in the promoter region of this gene, leading to transcriptional silence of the mRNA and absence of FMRP translation [1, 2]. Affected full mutation individuals have >200 pure CGG repeats. Premutation carriers have 55–200 CGG repeats with one AAG interruption or absent AGG interruption resulting in increasing length of pure CGG repeats at the 3′ end of the CGG repeat tracts. Normal individuals have 6–54 CGG repeats with two or more stabilizing AGG interruptions occurring once every 9 or 10 CGG repeat blocks [3, 4]. The common patterns are (CGG)9AGG and (CGG)10AGG, found in various populations. However, the (CGG)6AGG pattern (designated as 6A) has been reported exclusively in Asian populations [5–11], leading to the possibility that this 6A pattern may have originated in Asia.

To explore the evolution of the 6A pattern, we studied 176 unrelated Thai males with 19–56 CGG repeats using 8 SNPs near the CGG repeats of the* FMR1* gene. Of these 176 samples, we identified AGG interruption patterns from 95 samples with different CGG repeats using direct DNA sequencing. We found a specific SNP haplotype linked to the 6A pattern, and we also found something new that the SNP haplotypes showed strong associations between the common CGG repeat groups (29, 30, and 36) and AGG interruption patterns, suggesting different evolutionary lineages in the common CGG repeats of the* FMR1* gene.

## 2. Materials and Methods

### 2.1. DNA Samples

DNA was extracted from whole blood using the standard phenol/chloroform method. The PCR for the* CGG-FMR1* gene and methylation specific PCR were used with minor modification as previous reports [15, 16]. We selected 176 unrelated Thai males in this study, ranging from 19 to 56  CGG repeats. At this time the Thai population is known to have three common alleles, 29, 30, and 36 CGG repeats [15]. In the analysis, samples were divided into 6 groups corresponding to common and uncommon CGG repeats: 19–28, 29, 30, 31–35, 36, and 37–56. The study protocol was approved by the Institutional Ethics Committee.

### 2.2. SNP Study

**Figure 1:**
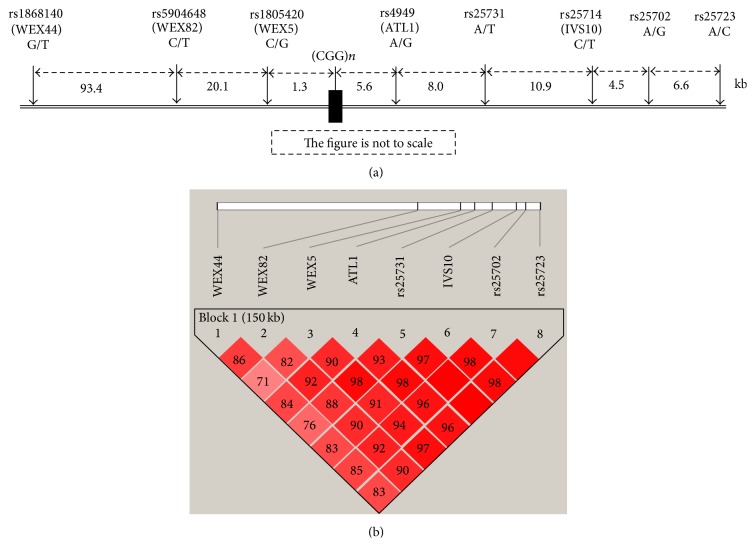
(a) The locations of the 8 SNPs. SNP-alleles of each locus are indicated under the SNPs. The distance between each SNP in Kb is shown below. The figure is not to scale. (b) Linkage disequilibrium (*D*′) plot of the 8 SNPs within 150 kb of the CGG-*FMR1* gene. All SNPs pairs have high *D*′ values, more than 80 or close to 80.

We selected 2 prior investigated SNPs, ATL1 or rs4949, IVS10 or rs25714 [17]. Six additional SNPs, WEX44 (rs1868140), WEX82 (rs5904648), WEX5 (rs1805420), rs25731, rs25702, and rs25723, were obtained from the previous reports [12, 13, 18]. The* FMR1* genomic and SNP position references were according to GenBank reference sequences L29074 and NC_000023.11. These SNPs are located both proximally and distally to the CGG repeats region of the* FMR1* gene (Figure 1(a)). Primer sequences and PCR conditions of all SNPs are shown in Table 1. A single-tube multiplex PCR was performed in a 10 *μ*L reaction containing 50 ng of genomic DNA, 1x PCR buffer, 200 *μ*M dNTPs, and 0.5 U Taq DNA polymerase (Invitrogen). The MgCl_2_ concentration and the presence or absence of an adjuvant in the PCR reactions were optimized to obtain the maximum yield of multiplex PCR products. In order to enhance the efficiency of allele-specific amplification, the concentration ratios of tetraprimer for each SNP assay were adjusted to produce a similar band intensity of each PCR product after gel electrophoresis. For the rs25731 SNP locus, PCR reactions were performed in a 20 *μ*L PCR reaction consisting of 100 ng of genomic DNA, 1x PCR buffer, 200 *μ*M dNTPs, 1.5 mM MgCl_2_, 0.25 *μ*M of each primer, and 1 U Taq DNA polymerase. The reactions were initially denatured for 5 min at 95°C, followed by 35 cycles of 30 sec at 95°C, 30 sec at appropriate annealing temperature, and 30 sec at 72°C and a final extension at 72°C for 10 min. Then 4 *μ*L of the rs25731 PCR reaction was digested with 4 units of* DraI*. Direct PCR products or digested PCR products were electrophoresed on 2.5% agarose gel and stained with ethidium bromide.

### 2.3. Sequencing Analysis of AGG Interruption Patterns

For accurate AGG interruption patterns, direct sequencing across the CGG repeats region was performed with primer A [1] and primer 571R [19] in a 50 *μ*L reaction volume comprised of 250 ng of genomic DNA, 50.25 mM Tris-HCl pH 8.8, 12.45 mM (NH_4_)_2_SO_4_, 1 mM MgCl_2_, 200 *μ*M dATP, 200 *μ*M dCTP, 200 *μ*M dTTP, 100 *μ*M dGTP, 100 *μ*M 7-deaza dGTP, 0.25 *μ*M of each primer, 10% DMSO, 128 *μ*g/mL BSA, and 2.5 units of Immolase DNA polymerase (Bioline). The PCR reactions were initially denatured for 9 min at 95°C, followed by 35 cycles of 1 min at 95°C, 1 min at 64°C, and 1 min at 72°C and a final extension at 72°C for 10 min. The PCR products were purified by a QIA quick PCR purification kit (Qiagen). Sequencing reactions were carried out in a 10 *μ*L reaction consisting of 1x BigDye terminator v1.1 ready reaction premix and 1.6 *μ*M of the internal sequencing primer FXS-SEQF (5′-TCTGAGCGGGCGGCGGGCCGA-3′) for forward reactions or primer 571R for reverse reactions. Cycle sequencing conditions were performed in a GeneAmp PCR System 9700 thermal cycler with a temperature profile of 1 min at 96°C followed by 25 cycles of 10 sec at 96°C and 4 min at 60°C. The sequencing products were purified to remove unincorporated fluorescent dye terminator using a DyeEx 2.0 spin kit (Qiagen). All sequencing pellets were dissolved with 15 *μ*L template suppressor reagent and separated by an ABI PRISM 310 genetic analyzer. The AGG interruption patterns were written in abbreviation, for example, 9A9A9, where 9 was (CGG)9 and A was AGG.

### 2.4. Data Analysis

The Haploview 4.2 program was used for SNP haplotypes analysis. We used Fisher's exact tests to examine the differences in haplotype frequencies among CGG repeat groups. The significant *P* value was assigned at 0.05.

## 3. Results

### 3.1. Haplotype Analysis

The high linkage disequilibrium found among the 8 SNPs studied is shown in Figure 1(b). Allele frequencies of all SNPs are shown in Table 2. When we analyzed the SNP haplotypes, three major haplotypes, GCGGATAA (Hap A), GCCGTTAA (Hap B), and TTCATCGC (Hap C), were found. The rare haplotypes (Hap D) included 11 different haplotypes with frequencies of less than 5% each. Hap A was similar to Hap B with 2 allele differences in the SNP loci (rs1805420 and rs25731) whereas Hap A was different from Hap C for all alleles in 8 SNPs.

### 3.2. Association of SNP Haplotypes and CGG Repeats

We divided the 176 samples into 6 groups based on the common and uncommon CGG repeats from small to large alleles (19–28, 29, 30, 31–35, 36, and 37–56) shown in Table 3. Strikingly, we found statistically significant associations between haplotypes and the common CGG repeat groups (Fisher's exact test < 0.001) but no statistical significance was found in other uncommon CGG repeat groups (Fisher's exact test = 0.0955). The 29-CGG-repeat group was associated with Hap A (41/55 or 74.5%), while the 30-CGG-repeat group was associated with Hap C (30/37 or 81.1%). In contrast, only one chromosome with Hap A and Hap C was observed in each of the 30- and 29-CGG-repeat groups. The 36-CGG-repeat group was associated with Hap B (27/32 or 84.4%). Hap B was not present in the 30-CGG-repeat group and only a few occurrences were noted in the 29-CGG-repeat group (5.5%). The large CGG repeat (37–56) group was related to Hap A or Hap B (12/15 or 80%), while the 19–28- and 31–35-CGG-repeat groups had 44.4% (8/18) and 31.6% (6/19) of Hap A and Hap B, respectively.

### 3.3. Association of SNP Haplotypes and AGG Interruption Patterns

**Figure 2:**
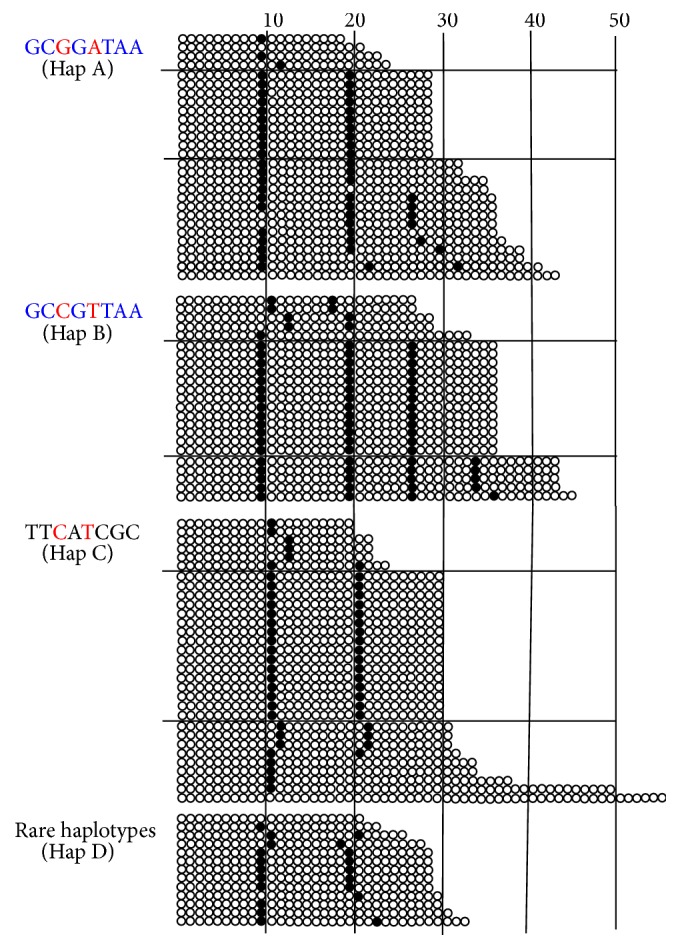
AGG interruption patterns of 95 X chromosomes. The CGG repeats are classified in haplotype groups. The AGG interruption patterns are shown from the 5′ to the 3′ ends of the CGG repeat tracts. A white circle represents a CGG and a black circle represents an AGG. The numbers of CGG repeats are indicated as numbers on top of the vertical lines (10, 20, 30, 40, and 50).

We randomly selected 95 X chromosomes from 176 samples (54%) for DNA sequencing, including uncommon and common alleles. The results revealed variety in both numbers of AGG and AGG interruption patterns in the CGG repeats of the* FMR1* gene (Figure 2). Most normal alleles had 2  AGG interruptions (48/95 or 50.5%). Alleles with a single or 3  AGG interruptions had the same frequencies of 20% (19/95). The no AGG and 4 AGG interruptions had frequencies of 4.2% (4/95) and 5.3% (5/95), respectively. The no AGG interruption was found in either low CGG repeats (21) or high CGG repeats (43 and 56) while the 4-AGG interruption was found in only high CGG repeats (43 and 45). The 3-AGG and 4-AGG interruptions were exclusively found in the Hap A and Hap B groups. However, no AGG and 2-AGG interruptions were found in all haplotypes. We also observed an allele possessing a 5′ tract with 20  CGG repeats (20A9). The 29 -CGG-repeat group with Hap A had an AGG configuration of 9A9A9 (10/17). The 30-CGG-repeat group with Hap C had an AGG configuration of 10A9A9 (16/18). The 36 CGG repeats with Hap B had an AGG configuration of 9A9A6A9 (13/18). This (CGG)6AGG pattern seemed specific to chromosomes with Hap B (i.e., 10A6A9 in 27  CGG repeats, 12A6A9 in 29 CGG repeats, 9A9A6A9 in 36  CGG repeats, 9A9A6A6A9 in 43 CGG repeats, and 9A9A6A8A9 in 45 CGG repeats). Only one chromosome with Hap B had the 9A23 pattern (33 CGG repeats) from 23 chromosomes with Hap B studied. Likewise, we observed that the 9A and 10A patterns at 5′ of the CGG repeats tract were related to Hap A and Hap C, respectively.

## 4. Discussion

The haplotype analysis using 8 SNPs in the present study provided more information than in previous studies [9, 17] which could not distinguish haplotypes with 29  CGG repeats from those with 36  CGG repeats (the third common allele exclusively found in Asians). Most chromosomes with 29 and 36  CGG repeats in Thai, Chinese, and Malay populations have G-T of the ATL1-IVS10 haplotype while the A-C haplotype was linked to chromosomes with 30  CGG repeats in Thai, Malay, Chinese, and Indian populations [9, 17]. Table 2 shows that the 29 and 36 CGG repeat groups had different haplotypes from two SNPs (rs1805420, rs25731).

Analysis of haplotypes using 8 SNPs in our study showed significant associations between haplotypes and the common CGG repeats (29, 30, and 36). The 29-CGG-repeat group was associated with haplotype GCGGATAA (Hap A), the 30-CGG-repeat group was associated with haplotype TTCATCGC (Hap C), and the 36-CGG-repeat group was associated with haplotype GCCGTTAA (Hap B). The uncommon CGG repeats of the 19–28, 31–35, and 37–56 groups were not associated with any haplotype and had similar distributions of haplotypes. These findings suggest that uncommon CGG repeats randomly occur in all three common and rare haplotypes.

**Figure 3:**
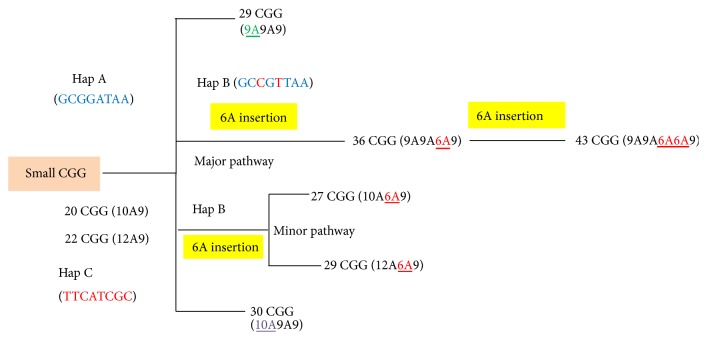
Simplified evolutionary pathways of the hypothesis. Chromosomes with 29 and 30 CGG repeats may have different evolutionary pathways since they had different haplotypes and AGG interruption patterns. The 6A pattern was linked to Hap B possibly derived from chromosomes with Hap A (major pathway) or Hap C (minor pathway).

Most of chromosomes with 36  CGG repeats and Hap B had an AGG configuration of 9A9A6A9 that might be derived from chromosomes with 29  CGG repeats and Hap A (9A9A9) by 6A insertion [5]. This formation was also found in chromosomes with 43  CGG repeats and Hap B (9A9A6A6A9), which might be derived from chromosomes with 36  CGG repeats and Hap B by 6A insertion (Figure 3). However, a few Hap B-chromosomes with 27 and 29  CGG repeats had AGG configurations of 10A6A9 and 12A6A9 that might be derived from 20 (10A9) and 22 (12A9) CGG repeats of chromosomes with Hap C by insertion of 6A pattern (Figure 3).

Hap A and Hap C had different alleles in all SNPs. This suggests that Hap A and Hap C may have different evolutionary pathways. However, Hap A and Hap B are likely evolutionarily derived since they had similar SNP haplotypes (Table 3) and both haplotypes carried 9A pattern at 5′ of the CGG repeats tract (Figures 2 and 3). The evolution of CGG repeats is likely from primitive small to large CGG repeats. An evolutionary study of the CGG repeats of the* FMR1* gene showed that most nonprimate mammals have a small number of uninterrupted CGG repeats with a mean of ~8 repeats, while the repeats of primates are larger with a mean of ~20 repeats and more highly specific interruptions [20]. Therefore, we hypothesize that there may be two distinct pathways in our findings. First, chromosomes with 29 and 30  CGG repeats may independently arise from Hap A and Hap C by gradual replication slippage or recombination via the smaller alleles [20] and were stable by the 9A9A9 and 10A9A9 patterns, respectively [11, 21]. Second, the 6A pattern was linked to chromosomes with Hap B possibly derived from chromosomes with Hap A (major pathway) or Hap C (minor pathway). Simplified pathways of the hypothesis are shown in Figure 3. In addition, perhaps the 6A pattern enhances the stability of CGG repeat tracts [22, 23]. Thus, chromosomes with 36  CGG repeats linked to the 6A pattern have become the third most common allele in only Asian populations. It is also relevant to note that, to date, the 6A pattern has been exclusively found in Asians [5–11]. A study based on an Eskimo population indicated that the 6A pattern has been stably conserved for 15,000–30,000 years, since this group migrated from Asia to North America [7].

It has been proposed that AGG interruptions play a crucial role in maintaining the stability of the CGG repeats since premutation alleles often contain only one AGG or no AGG interruptions [3, 4, 24–26]. Haplotypes analysis using microsatellites near the* FMR1* gene (DXS548-FRAXAC1-FRAXAC2) found that specific haplotypes were associated with the loss of AGG interruptions of the CGG repeats in Caucasians [27] and Jewish Tunisians [28]. In contrast, the findings in African Americans using those three microsatellites and the SNP, ATL1 did not show a haplotype association with CGG repeats instability [29]. Also, our findings in this study support earlier studies where the SNP haplotype association between nearby SNPs and AGG interruption patterns in CGG repeats of the* FMR1* gene likely reflects linkage disequilibrium in each population [9, 17, 30]. Therefore, it is difficult to determine if an associated haplotype is a real factor for CGG repeats instability or a linkage disequilibrium in a specific population [31].

## 5. Conclusion

Our study showed new evidence that the specific haplotype (Hap B) was strongly linked to the 6A pattern in Thai subjects since almost all chromosomes with Hap B had at least one 6A configuration, regardless of CGG repeats (i.e., 10A6A9, 12A6A9, 9A9A6A6A9, and 9A9A9A6A8A9). The 6A pattern and Hap B may have originally occurred in the ancestors of Asian populations. However, we could not completely exclude that the findings may be by chance or sample selection bias. Further studies of SNP haplotypes and AGG interruption patterns in other Asian populations would be warranted, to confirm and expand on our findings.

## Figures and Tables

**Table 1:** The oligonucleotide sequences of primers and the PCR conditions.

Locus	Name	Sequence (5′ to 3′)	References	Conc. (*μ*M)	MgCl_2_ Conc. (mM)	Adjuvant (Conc.)	Annealing temperature (°C)	Product size (bp.)
WEX44 (rs1868140)	WEX44F WEX44R WEX44TF WEX44GR	CTATCTGGGGGCAAATGAACCATAG CTCTGAGTTTACGCTCCCCAG CCTAACAGTATAGACCATGATGGAAACATAT CAGAGAATAGTTTCAGTTTCTCAGTTTAAACTC	This study This study Ennis et al. (2007) [12] Ennis et al. (2007) [12]	0.1 0.1 1.5 1.5	1.5	Q-solution (1x)	57	Control (333) T allele (134) G allele (262)

WEX82 (rs5904648)	WEX82F WEX82R WEX82CF WEX82TR	GACAACCCATAATCTGTCATTGG CCACCAGTACTTCCTAATGATA TATAACCATGTAAAAAGATCTTCAATC CCTCTGATTATTAATTTATTAATGCA	This study This study This study This study	0.08 0.08 3 7.84	2.5	Betaine (0.6 M)	62	Control (398) C allele (152) T allele (295)

WEX5 (rs1805420)	WEX5F WEX5R WEX5CF WEX5GR	GAATGTGGCCCTAGATCCAC GTGCTAACGAGAAATCGGTG CTTATCACAGCTGCAACTACAC CAAATTGTCAGACAAGTAAACC	This study This study This study This study	0.1 0.1 1 3	1.25	BSA (0.13 mg/mL)	60	Control (361) C allele (143) G allele (261)

ATL1 (rs4949)	ATL1F ATL1R ATL1AF ATL1GR2^*∗*^	ACCCTGATGAAGAACTTGTATCTCT GAAATTACACACATAGGTGGCACT TGTACATTTTCCAAATGCAAAGA AGAGACACAGAATCATAAATGC	Brightwell et al. (2002) [13] ^*∗*^Modified	0.1 0.1 3 0.1	1.5	BSA (0.13 mg/mL)	53	Control (302) A allele (107) G allele (239)

rs25731	731F 731R	AGATTCCCACCTCCTGTAGG CATGCTCTGAGTACTGCTC	This study This study	0.25 0.25	1.5	—	60	Product (269) Cut by *DraI* A allele (125, 119, 25) T allele (150, 119)

IVS10 (rs25714)	IVS10F IVS10R IVS10TF IVS10CR	AAAGCTGATTCAGGAGATTGTG ACTGCATTAGAGGACAGAGA CAAGAAGAGGTATGTTACAGTAT TTATTATATGTGCCACAAAATATTGG	This study Xu et al. (1999) [14] This study This study	0.1 0.1 3 3	1.5	BSA (0.13 mg/mL)	53	Control (268) T allele (189) C allele (127)

rs25702	702F 702R 702AF 702GR	ACTCAGTTTAGGCAATCCTG CACAGCTAGTTCATTTGCTG TCAGTTTAGTTAGTGTGATGTA GAAATTTTAAGGAGGCATAATC	This study This study This study This study	0.15 0.15 3 2.8	1.5	BSA (0.13 mg/mL)	55	Control (379) A allele (148) G allele (274)

rs25723	723F 723R 723AF 723CR	GAGCGAGACTGTCTGGGAA TGGAAGGACTGGAATCCTAG ACATTTAAAACACATGCACATATA TTTCAAAGTATGTTTAAGTAGTAG	This study This study This study This study	0.05 0.05 0.6 4	1.5	BSA (0.13 mg/mL)	55	Control (330) A allele (263) C allele (114)

**Table 2:** The allele frequencies of the 8 SNPs studied.

SNP	Major allele (%)	Minor allele (%)
WEX44 (rs1868140)	G (65.9)	T (34.1)
WEX82 (rs5904648)	C (65.3)	T (34.7)
WEX5 (rs1805420)	C (57.4)	G (42.6)
ATL1 (rs4949)	G (67.0)	A (33.0)
rs25731	T (58.0)	A (42.0)
IVS10 (rs25714)	T (64.2)	C (35.8)
rs25702	A (64.2)	G (35.8)
rs25723	A (64.8)	C (35.2)

**Table 3:** SNP haplotypes frequencies in different CGG repeat groups.

Haplotype	Frequencies of the CGG groups (number)						
	19–28 CGG^**∗∗**^ (18)	29 CGG^**∗**^ (55)	30 CGG^**∗**^ (37)	31–35 CGG^**∗∗**^ (19)	36 CGG^**∗**^ (32)	37–56 CGG^**∗∗**^ (15)	Total number (176)
GCGGATAA (Hap A)	0.278 (5)	*0.745* * (41)*	0.027 (1)	0.263 (5)	0.156 (5)	0.467 (7)	**0.364** **(64)**
GCCGTTAA (Hap B)	0.167 (3)	0.055 (3)	0	0.053 (1)	*0.844* * (27)*	0.333 (5)	**0.222** ** (39)**
TTCATCGC (Hap C)	0.333 (6)	0.018 (1)	*0.811* * (30)*	0.526 (10)	0	0.200 (3)	**0.284** ** (50)**
Rare haplotypes (Hap D)	0.222 (4)	0.182 (10)	0.162 (6)	0.158 (3)	0	0	**0.130** **(23)**

Comparison based on CGG repeats groups.

^**∗**^Common CGG repeat groups (29, 30, and 36; Fisher's exact test; *P* value < 0.001; statistical significance).

^**∗∗**^Uncommon CGG repeat groups (19–28, 31–35, and 37–56; Fisher's exact test; *P* value = 0.0955; no statistical significance).
